# Challengeable Legislation Against Fatal Occupational Accidents in Republic of Korea: Serious Accidents Punishment Act of Korea

**DOI:** 10.1016/j.shaw.2022.01.005

**Published:** 2022-01-21

**Authors:** Seong-Kyu Kang

**Affiliations:** Gil Medical Center, Gachon University College of Medicine, 21 Namdongdae-ro 774-beon gil, Namdong-gu, Incheon, 21565, Republic of Korea

**Keywords:** Fatal occupational accidents, Serious Accidents Punishment Act, Occupational injuries, Korea

The fatal occupational accident (injury) rate in Republic of Korea remains higher than that in the European Union (EU) countries, although there has been great success in preventing occupational accidents in the last two decades [1]. In 2021, there were 828 deaths caused by occupational accidents among approximately 19 million workers who registered for Workers' Compensation Insurance (Table 1). The fatal accident rate in 2021 was 4.31 per 100,000 workers, while that of the EU 15 countries was 1.46 in 2018 [2].

**Table 1:** Fatal occupational accidents and fatility rates by year.

Year	Number of workers	Fatal cases	Fatality rate
2000	9,485,557	1,476	15.56
2001	10,581,186	1,551	14.66
2002	10,571,279	1,378	13.04
2003	10,599,345	1,533	14.46
2004	10,473,090	1,537	14.68
2005	11,059,123	1,398	12.64
2006	11,688,797	1,332	11.40
2007	12,528,879	1,383	11.04
2008	13,489,986	1,448	10.73
2009	13,884,927	1,401	10.09
2010	14,198,748	1,383	9.74
2011	14,362,372	1,129	7.86
2012	15,548,423	1,134	7.29
2013	15,449,228	1,090	7.06
2014	17,062,308	992	5.81
2015	17,966,981	955	5.32
2016	18,431,716	969	5.26
2017	18,560,142	964	5.19
2018	19,073,438	971	5.09
2019	18,725,160	855	4.57
2020	18,974,513	882	4.65
2021	19,228,561	828	4.31

The fatal accident rates were higher than 10 per 100,000 workers until 2010; however, this included some fatal cases unrelated to any circumstances controlled by employers, such as fatalities during commuting or sports-related activities. After excluding fatalities caused by non-direct occupational activities, fatal occupational accident rates in Republic of Korea dropped to 7.86 in 2011 (Fig. 1). However, fatal occupational accidents are three times higher than in the EU 15 countries, which mostly stem from traditional causes like unsafe facilities and dangerous equipment. In 2021, the causes of fatalities were falls from heights (295), caught in objects (77), collisions (55), accidents by moving vehicles (51), overturned (48), hit by objects (42), explosion or fire (33), collapse (26), fall on the same level (15), etc.

**Fig. 1:**
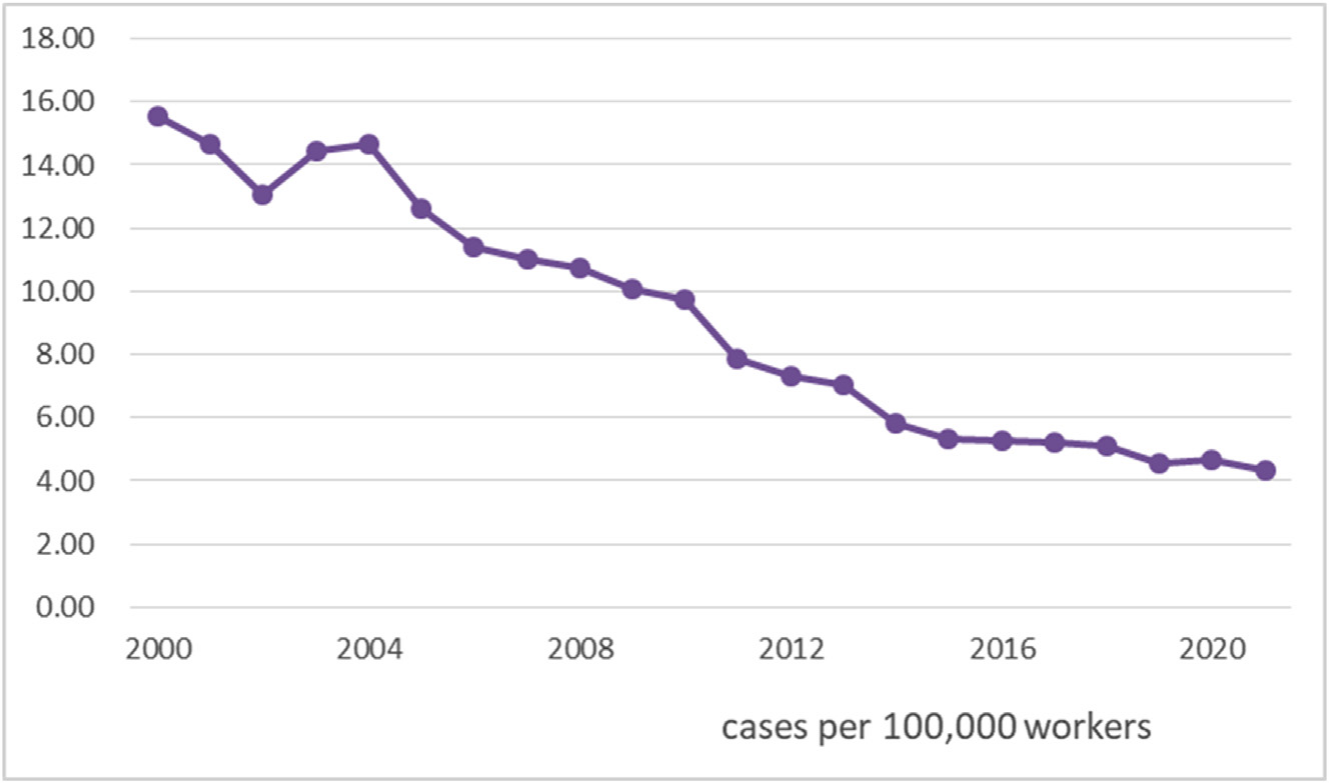
Occupational accident fatality rates by year in Korea.

In 2019, a maintenance worker at a subcontractor of the KEPCO (Korea Electric Power Corporation) was killed while employed to maintain conveyor belts in a coal-fired power plant. Unlike many other previous occupational deaths, his death garnered enormous public attention [3] due to the public's elevated attention to safety issues provoked by the tragic Sewol ferry disaster, which resulted in more than 300 deaths and missing students in 2014. Heavy public demand pushed the parliament to completely amend the Occupational Safety and Health Act in 2020 in terms of definitions, object, work contracts, safety standards, punishments, etc.

However, on April 29, 2020, another tragic fire accident occurred at an under-construction cold storage warehouse, which killed 38 workers due to asphyxia and burns. This was a repeated incident that occurred in a cold warehouse under construction; a same accident in 2008 had 40 fatalities. However, the warehouses owner and the builder received no punishment or even minimal penalties because the deceased workers were contractors or subcontractors, not their own employees.

The resulting public anger pressed the government and parliament to establish strong punishment legislation. The new act, the Serious Accidents Punishment Act, was established in 2021 [4]. According to this law, employers shall be imprisoned for more than one year if a fatal occupational accident occurs in their workplace, including contractors. The Act adopted the Corporate Manslaughter and Corporate Homicide Act of the United Kingdom (UK). The differences between the two laws include the subject of punishment and the causes of death.

The Korean act can punish both employers and corporations, while the UK law punishes only corporations. The Korean act also covers deaths caused by occupational diseases like acute chemical poisonings, and even includes those caused by occupational cancers and cerebro-cardiovascular diseases.

Furthermore, a problem arises out of the different legal systems in Republic of Korea and the UK. The legal system in the Republic of Korea is based on the continental law system, which originated from German law or civil law. The UK, by contrast, has a common law system. One main difference between continental and English law is the target of regulation.

The English system allows employers to choose the option of prevention, and they shall be punished only if accidents happen without proper management. When accidents occur, the jury applies common sense standard regarding accident prevention rather than using detailed regulations and standards.

By contrast, Korean law, like continental law, stipulates every rule and regulation and punishes employers if they are violated. Continental law punishment is based on the “principle of legality.” Employers who are guilty of occupational accidents shall be punished only under the law, which means the punishment may not consider the cause of the accident, but only follows the regulations that govern employers. Therefore, employers who may have a fatal accident, may not be punished based on the accident itself, but based on the regulations and standards they had to follow. In this continental legal system, an inspection strategy would be effective in decreasing occupational fatalities if it focuses on the major causes of accidents; however, the accident investigation focused on determining the number of violations committed. This may explain why the number of occupational fatalities in Republic of Korea are higher than those in EU countries.

Another problem arises from the cause of death. The cases of fatal occupational accidents are covered under workers' compensation, which is generous in accepting claims because it is viewed as part of the social security system in Republic of Korea. Therefore, the fatal occupational accidents accepted by this system include those caused by employers' fault or negligence, as well as no fault, including even welfare concepts.

The Serious Accidents Punishment Act stipulates that an employer shall be punished for more than one year in prison if an occupational death has occurred and if there was no proper occupational health and safety plan in place. The Employers' Association has complained about the vagueness of the properness standard in evaluating the performance of accident prevention plans and practices. In addition, the trade unions complain about three years of delayed enforcement for enterprises with less than 50 employees, where most fatalities occur.

This new challenging act has been put into practice, and without a doubt, it will decrease fatal occupational accidents to a certain extent. However, decreasing fatalities to the level of European countries will be difficult without changing the Ministry of Employment and Labor's inspection strategy for occupational safety and health.

## Conflicts of interest

The author declares that there is no conflict of interest.
